# Shrinking Lung Syndrome in a Systemic Lupus Erythematous Patient Improved by Rituximab: A Case Report With Literature Review

**DOI:** 10.7759/cureus.50229

**Published:** 2023-12-09

**Authors:** Layth Al-Karaja, Fatima O Alshayeb, Dana Amro, Yazan F khdour, Laith Alamlih

**Affiliations:** 1 Internal Medicine, Al-Quds University, Jerusalem, PSE; 2 General Practice, Jordan University of Science and Technology, Amman, JOR; 3 Allergy and Immunology, Jordan University of Science and Technology, Amman, JOR; 4 Rheumatology, Princess Alia Governmental Hospital, Hebron, PSE; 5 Rheumatology, Hebron University, Hebron, PSE

**Keywords:** rheumatology & autoimmune diseases, case report, rituximab, systemic lupus erythematous, shrinking lung syndrome

## Abstract

Shrinking lung syndrome (SLS) is a rare complication of autoimmune and connective tissue diseases like systemic lupus erythematosus (SLE). A 35-year-old female patient, diagnosed with SLE, came to the hospital complaining of severe dyspnea and pleuritic pain for several months that was worsening on exertion. Imaging (X-ray and CT scan) of the chest at the time of presentation showed bilateral basal atelectasis with elevated diaphragm. Pulmonary function test (PFT) showed restrictive findings including forced expiratory volume in the first second (FEV1) of 37%, total lung capacity of 40%, and vital capacity of 32% predicted with a restrictive pattern on flow volume loop confirming the diagnosis of SLS. The treatment focused on methotrexate and rituximab. Patients with a known history of SLE who start respiratory symptoms like cough and dyspnea should be ruled out of SLS at the earliest as it can be deadly in the later stages.

## Introduction

Systemic lupus erythematosus (SLE) is an autoimmune disease, causing widespread inflammation and tissue damage, with multisystemic involvement. It can affect the joints, lungs, skin, kidneys, brain, and blood vessels [1]. The disease has several phenotypes, with varying clinical manifestations in patients ranging from mild mucocutaneous manifestations to multiorgan failure and severe central nervous system involvement, and it has multifactorial etiology, variable prognosis, and multiple circulating autoantibodies. 

The prominent SLE feature is the production of multiple circulating autoantibodies such as double-stranded DNA (dsDNA), Smith antigen antibodies, which are specific serologic markers of SLE, and anti-nuclear antibodies (ANAs), which are considered markers of diagnosis and prognosis of SLE [2,3].

Shrinking lung syndrome (SLS) is a rare complication of autoimmune and connective tissue diseases like SLE, polymyositis, and Sjögren syndrome. SLS was described for the first time in 1965, with a prevalence of around 1%, but reported in up to 6% of severe SLE cases. SLS is more common in females [4-7].

SLS shows a restrictive pattern in pulmonary function tests (PFTs) with a reduction in forced vital capacity (FVC), lung volumes which commonly show a decline of forced expiratory volume in one second (FEV1), lung diffusion capacity for carbon monoxide (DLCO), and total lung capacity (TLC). Patients usually present with progressive exertional dyspnea as the main concern, accompanied by pleuritic chest pain in 80% of cases, and less frequently by cough [8,9].

Here, we report a case of a 35-year-old female with SLS as a complication of SLE.

## Case presentation

**Figure 1:**
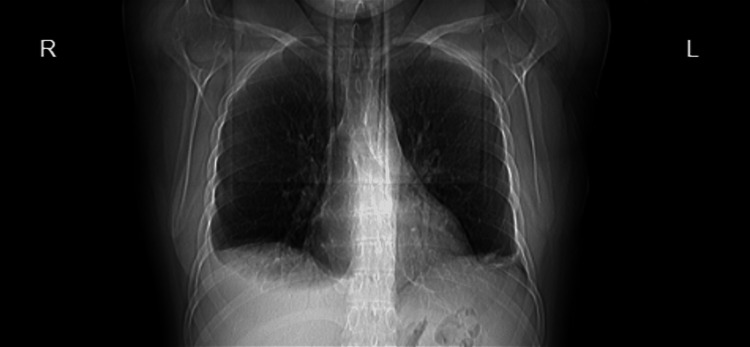
Bilateral atelectasis with elevated diaphragm.

A 35-year-old female patient, who was previously diagnosed with SLE, presented to the hospital complaining of severe dyspnea and pleuritic pain that had worsened over several months, especially during exertion. Imaging tests, including X-ray and CT scan, showed bilateral basal atelectasis with an elevated diaphragm, as seen in Figure 1. PFT confirmed a restrictive pattern on the flow volume loop, with findings that included FEV1 of 37%, TLC of 40%, and vital capacity (VC) of 32%, which led to a diagnosis of SLS.

As per her medical history, the patient first presented with multiple joint pains and blistering on the inner thigh, which later progressed to hair loss. She was initially suspected to have rheumatoid arthritis (RA) and was started on methotrexate (MTX) and prednisone (5 mg), with no improvement and a persistently elevated erythrocyte sedimentation rate (ESR). After eight years, her left knee pain became so severe that she could not move it, and was prescribed non-steroidal anti-inflammatory drugs (NSAIDs) by another physician. However, she later experienced acute abdominal pain with nausea, vomiting, and low-grade fever, suggestive of NSAID-induced gastritis. During the same hospitalization, further testing revealed pericardial effusion, and pericardiocentesis yielded around 80 ml of thick bloody fluid. Cytology of the pericardial fluid confirmed the diagnosis of SLE, instead of RA. The treatment regimen was changed, and prednisone was tapered and eventually withdrawn after around 10 years of intake.

Currently, the patient is on a treatment regimen of rituximab (once every six months) and hydroxychloroquine, and her dyspnea has improved since her initial presentation. A follow-up PFT showed significant improvement, and she is scheduled for another PFT. Furthermore, she is seeing a psychiatrist via telemedicine for anxiety and depression, which developed due to the years of being undiagnosed.

## Discussion

SLS is an infrequent complication of SLE with prevalence of 0.5-1.1%. Although the prevalence is low, it happens to be the first manifestation of SLE in 9.5% of the presented patients.

The exact pathogenesis of SLS has been unknown although this syndrome was recognized more than 50 years ago. Most cases of SLS have been reported with SLE and recently it started appearing with Sjogren’s syndrome [10]. However, no associations were found relating this syndrome with other connective tissue diseases.

Many authors in the literature concluded that SLS is a heterogeneous syndrome with relation to many pathogenic mechanisms. One of the many theories shows chest wall restriction involvement, while another theory suggests diaphragmatic involvement. Studies have shown abnormalities in diaphragmatic pressure related to its dysfunction, which was thought to contribute to the decrease in lung volume [11-14].

Despite the number of theories made, some could not demonstrate any abnormalities regarding the diaphragm [15]. Although accumulated evidence does not support these hypotheses, other theories included chest muscle myopathy secondary to SLE, phrenic nerve involvement, and any generalized muscle weakness [15,16].

The presence of demyelinating neuropathy or axonal degeneration because of diaphragmatic weaknesses was excluded by electromyographic studies of the phrenic nerve [16]. It was reported by both Ciaffi et al. and Laroche et al. that the presence of bilateral phrenic nerve paralysis is a cause of SLS in patients with SLE [8,15]. Phrenic nerve palsy might be more reasonably attributed to peripheral neuropathy linked to SLE rather than SLS. In studies by Hardy et al. and Shin et al., the patients however did not have a history of long-term steroid use, so myopathy related to steroids has been ruled out. Muscle atrophy is prednisone dose-dependent [17,18]. In most cases with the use of high doses of corticosteroids, there was rapid improvement. Another possibility is anti-malarial-induced myopathy, although it is associated with high doses of hydroxychloroquine, in levels not normally given for RA or SLE. In these conditions, cardiotoxicity and ocular side effects are usually seen before the myopathy. On muscle biopsy, typical vacuolar changes can be seen in these patients [19,20]. Therefore, antimalarial therapy is unlikely to cause diaphragmatic weakness.

Markedly thin hemidiaphragms with diffuse fibrotic changes are the only reported clinical diagnostic studies [18]. For this rare condition, these findings help support the hypothesis of extrapulmonary restrictive etiology.

Although SLS presenting as the first manifestation is rare, it has been mentioned in a few cases but it usually presents in the later stages of SLE [4,17,21,22]. Patients usually complain at the presentation of exertional dyspnea, which may later progress to dyspnea at rest and pleuritic chest pain. Fever and cough are rarely present. While myositis has been documented in only 13% of cases, other features of underlying SLE may be present. The patient is usually tachypneic with rapid shallow breathing and accessory muscle usage can be noticed. Although there are no specific laboratory tests for SLS, elevated ESR and positive ANA were common findings in all cases. In imaging, chest X-ray shows thickening of pleura with elevation of hemidiaphragms or presence of pleural effusions. Usually, PFT shows a restrictive pattern, and blood gas results can be normal or with mild hypoxemia, especially at rest, which usually worsens with exertion [23].

Although there is no proven treatment, corticosteroids were found to be the most common agent used regardless of the different hypotheses about the pathogenesis [23-25]. The dose is tapered rapidly after the control of the signs and symptoms in cases treated successfully with corticosteroids. In addition to steroids, some immunosuppressive drugs, like azathioprine, hydrochloroquine, and cyclophosphamide, were used in the treatment of some cases. Although these medications are not clearly known to be useful in the management of SLS, some reports showed cases where corticosteroids failed while response was achieved from adrenergic agonists [26]. These reports postulated that there is an improvement in respiratory muscle strength because of the positive isotopic effect of the diaphragm, which resulted in increased pressure of transdiaphragm, noticed after adrenergic agonists’ therapy [27,28]. Theophylline, which was used in treatment by Van Veen et al., showed an increase in muscle strength of the diaphragm, which helped in the improvement of respiratory force [29]. The therapeutic effect reported in this patient could be due to the use of theophylline alone although he was treated previously with prednisone and azathioprine, but the effects of combination therapy cannot be ruled out. Noninvasive mechanical ventilation (bilevel positive airway pressure (BiPAP)) and oxygen supplementation are important in relieving hypoxemia in severe restrictive disease patients.

## Conclusions

With very few cases reported of SLS, confirming the best therapy is quite challenging. On the bright side, most patients show remarkable improvement in their lung functions over a period of time. So, it is safe to say that the prognosis of SLS in SLE is good. However, with the challenges present and late diagnoses made, SLS should be ruled out of SLE patients presenting with respiratory symptoms. Despite the rare occurrence of SLS, its presence in the late stages in SLE patients could have a bad prognosis.

## References

[1] Justiz Vaillant AA, Goyal A, Varacallo M (2023). Systemic lupus erythematosus. StatPearls [Internet].

[2] Dema B, Charles N (2016). Autoantibodies in SLE: specificities, isotypes and receptors. Antibodies (Basel).

[3] Yaniv G, Twig G, Shor DB (2015). A volcanic explosion of autoantibodies in systemic lupus erythematosus: a diversity of 180 different antibodies found in SLE patients. Autoimmun Rev.

[4] Hoffbrand BI, Beck ER (1965). “Unexplained” dyspnoea and shrinking lungs in systemic lupus erythematosus. Br Med J.

[5] Borrell H, Narváez J, Alegre JJ (2016). Shrinking lung syndrome in systemic lupus erythematosus: a case series and review of the literature. Medicine (Baltimore).

[6] Deeb M, Tselios K, Gladman DD, Su J, Urowitz MB (2018). Shrinking lung syndrome in systemic lupus erythematosus: a single-centre experience. Lupus.

[7] Duron L, Cohen-Aubart F, Diot E (2016). Shrinking lung syndrome associated with systemic lupus erythematosus: a multicenter collaborative study of 15 new cases and a review of the 155 cases in the literature focusing on treatment response and long-term outcomes. Autoimmun Rev.

[8] Ciaffi J, Gegenava M, Ninaber MK, Huizinga TW (2021). Shrinking lung syndrome: diagnostic and therapeutic challenges in 3 patients with systemic lupus erythematosus. J Clin Rheumatol.

[9] Colquhoun M, Akram S (2022). Shrinking lung syndrome. StatPearls [Internet].

[10] Tavoni A, Vitali C, Cirigliano G (1999). Shrinking lung in primary Sjogren’s syndrome. Arthritis Rheum.

[11] Gibson CJ, Edmonds JP, Hughes GR (1977). Diaphragm function and lung involvement in systemic lupus erythematosus. Am J Med.

[12] Martens J, Demedts M, Vanmeenen MT (1983). Respiratory muscle dysfunction in systemic lupus erythematosus. Chest.

[13] Thompson PJ, Dhillon DP, Ledingham J, Turner-Warwick M (1985). Shrinking lungs, diaphragmatic dysfunction, and systemic lupus erythematosus. Am Rev Respir Dis.

[14] Jacobelli S, Moreno R, Massardo L, Rivero S, Lisboa C (1985). Inspiratory muscle dysfunction and unexplained dyspnea in systemic lupus erythematosus. Arthritis Rheum.

[15] Laroche CM, Mulvey DA, Hawkins PN, Walport MJ, Strickland B, Moxham J, Green M (1989). Diaphragm strength in the shrinking lung syndrome of systemic lupus erythematosus. Q J Med.

[16] Wilcox PG, Stein HB, Clarke SD, Paré PD, Pardy RL (1988). Phrenic nerve function in patients with diaphragmatic weakness and systemic lupus erythematosus. Chest.

[17] Hardy K, Herry I, Attali V, Cadranel J, Similowski T (2001). Bilateral phrenic paralysis in a patient with systemic lupus erythematosus. Chest.

[18] Shin YS, Fink H, Khiroya R, Ibebunjo C, Martyn J (2000). Prednisolone-induced muscle dysfunction is caused more by atrophy than by altered acetylcholine receptor expression. Anesth Analg.

[19] Hughes JT, Esiri M, Oxbury JM, Whitty CW (1971). Chloroquine myopathy. Q J Med.

[20] Chapman RS, Ewen SW (1969). Chloroquine-induced myopathy. Br J Dermatol.

[21] Rubin LA, Urowitz MB (1983). Shrinking lung syndrome in SLE--a clinical pathologic study. J Rheumatol.

[22] Oud KT, Bresser P, ten Berge RJ, Jonkers RE (2005). The shrinking lung syndrome in systemic lupus erythematosus: improvement with corticosteroid therapy. Lupus.

[23] Warrington KJ, Moder KG, Brutinel WM (2000). The shrinking lungs syndrome in systemic lupus erythematosus. Mayo Clin Proc.

[24] Kitamura Y, Okano Y (1996). A case of the “shrinking lung syndrome” in SLE: improvement with corticosteroid therapy. Jpn J Clin Immunol.

[25] Walz-Leblanc BA, Urowitz MB, Gladman DD (1992). The “shrinking lungs syndrome” in systemic lupus erythematosus: improvement with corticosteroid therapy. J Rheumatol.

[26] Soubrier M, Dubost JJ, Piette JC (1995). Shrinking lung syndrome in systemic lupus erythematosus. A report of three cases. Rev Rhum Engl Ed.

[27] Muñoz-Rodríguez FJ, Font J, Badia JR, Miret C, Barberà JA, Cervera R, Ingelmo M (1997). Shrinking lungs syndrome in systemic lupus erythematosus: improvement with inhaled beta-agonist therapy. Lupus.

[28] Aubier M, Viires N, Murciano D, Medrano G, Lecocguic Y, Pariente R (1984). Effects and mechanism of action of terbutaline on diaphragmatic contractility and fatigue. J Appl Physiol Respir Environ Exerc Physiol.

[29] Van Veen S, Peeters AJ, Sterk PJ, Breedveld FC (1993). The "shrinking lung syndrome" in SLE, treatment with theophylline. Clin Rheumatol.

